# When Copper Meets Autoimmunity: A Rare Coexistence of Wilson's Disease and Systemic Lupus Erythematosus

**DOI:** 10.1002/ccr3.70159

**Published:** 2025-02-19

**Authors:** Suresh Bhagoowani, Uooja Devi, Aqsa Munir, Ummulkiram Hasnain, Saad Khan, Javed Iqbal, Tehseen Akhtar

**Affiliations:** ^1^ Department of Medicine II Dr. Ruth K. M. Pfau Civil Hospital Karachi Sindh Pakistan; ^2^ Dow University of Health Sciences Karachi Sindh Pakistan; ^3^ Saidu Medical College Karachi Pakistan; ^4^ Nursing Department Communicable Diseases Center Hamad Medical Corporation Doha Qatar

**Keywords:** autoimmune disorders, diagnosis, systemic lupus erythematosus, treatment, Wilson's disease

## Abstract

The coexistence of systemic lupus erythematosus (SLE) and Wilson's disease (WD) is exceedingly rare, posing significant diagnostic challenges due to overlapping clinical presentations. Neurological and psychiatric manifestations commonly associated with WD can obscure or mimic symptoms of other systemic diseases, complicating accurate diagnosis and management. We report the case of a 17‐year‐old female diagnosed with WD and concurrent SLE, treated at Civil Hospital, Karachi, Pakistan. The patient initially presented with neuropsychiatric symptoms typical of WD, confirmed by diagnostic findings indicative of hepatic copper accumulation. She later developed significant proteinuria, hematuria, fever, and positive autoimmune serologies, raising suspicion of concurrent SLE. Despite therapeutic interventions, including methylprednisolone (1 mg), the patient's condition deteriorated, and she unfortunately succumbed to complications from a blood transfusion reaction. This case underscores the importance of heightened clinical vigilance when managing overlapping presentations of WD and systemic autoimmune disorders like SLE. Early recognition of WD‐specific neurological and psychiatric symptoms is critical for timely diagnosis and intervention, potentially mitigating the risk of severe complications and poor outcomes.

**Summary**

- This case highlights the rare co‐occurrence of Wilson's disease and systemic lupus erythematosus (SLE), which complicates diagnosis due to overlapping symptoms.
- Treatment adjustments confirmed the SLE diagnosis, but the patient ultimately died from complications related to a blood transfusion reaction.
- The case emphasizes the need for careful diagnosis and management in complex systemic diseases.

## 1 Introduction

Wilson's disease (WD) is a genetic disorder caused by mutations in the ATP7B gene on chromosome 13, resulting in an autosomal recessive ailment. The poor excretion of copper by the biliary system is the primary anomaly associated with WD [1]. In Wilson's illness, there is a gradual rise in copper levels in the liver, eventually leading to an overflow, causing a multi‐organ involvement, including neurological and renal abnormalities. Dysfunction of ATP7B and hepatic dysfunction may also result in the buildup of other metals, like iron and manganese, within an individual's brain, possibly exacerbating symptoms of Wilson's illness [1]. Systemic lupus erythematosus (SLE) is an autoimmune condition that is more common in females and spares no organ. Organ damage manifests as immune system abnormalities and environmental, hormonal, and hereditary factors. The wide range of clinical symptoms includes manifestations in the skin and mucous membranes, musculoskeletal system, blood, heart and lungs, kidneys, and central nervous system. Lupus nephritis and neuropsychiatric lupus are regarded as the most severe types of organ involvement and can lead to a considerably shortened lifespan [2].

Seldom reports of SLE exacerbated by WD or the coexistence of two illnesses have been made. Here, we report a case of a young female patient suffering from both Wilson and SLE. A summary of Patients with WD and SLE from the Literature Review are presented in Table 1.

**TABLE 1:** Summary of patients with Wilson's disease and systemic lupus erythematosus (SLE) from literature review.

S.no	Study	Year	Age/Sex	Clinical features	Investigations	Management	Prognosis
1.	Lishan Xu et al. [3]	2021	35‐year‐old female	Asymptomatic with known SLE, Routine follow‐up revealed concerning findings on liver ultrasound.	Albumin: 33 g/L Other LFTs: Normal Ceruloplasmin: < 0.1 g/L Serum copper: 2.44 mol/L 24‐h urine copper levels: 110 g/24 h KF ring: Seen on slit‐lamp examination Low complement levels and positive antibodies for ANA and Anti‐dsDNA Abdominal ultrasound and CT: Suggestive of liver cirrhosis	Zinc sulfate, methylprednisolone and hydroxychloroquine	US and labs showed marked improvement on the 11th‐month follow‐up. Ceruloplasmin levels raised to 0.20 g/L.
2.	Yang et al. [4]	2024	9‐year‐old female	Hematuria	LFTs: Deranged Albumin: 25.7 g/L Ceruloplasmin: < 9.5 mg/dL Low complement levels, ANA, and anti‐dsDNA: Positive Abdominal US: Normal Kidney Biopsy: Positive for antibodies Tumor markers: Negative Liver biopsy: Findings of fibrosis, and cirrhosis	Liver transplantation	The patient died 4 days post‐surgery
3.	Zhang et al. [5]	2018	18‐year‐old female	Impaired speech and unusual limb movements for 2 years	LFTs: Deranged Albumin: 32 g/L Ceruloplasmin: 0.033 g/L KF ring: Seen on slit‐lamp examination MRI Head: anomalous signals in the thalamus and basal ganglia. ANA, anti‐SSA antibody, anti‐rRNP antibody: raised CT abdomen: widespread hepatic lesions with splenic and liver enlargement	IV Zinc sulfate and IV sodium dimercaptopropane sulfonate. Oral hydroxychloroquine, aspirin, and IV methylprednisolone. 1 month later, steroids decreased, and oral drugs replaced IV ones.	At 1 month follow‐up. neurological symptoms and labs significantly improved Her symptoms did, however, return 6 months later
4.	Hadef et al. [6]	2021	12‐year‐old male	Pale skin, yellow sclera and, Haematuria	LFTs deranged Ceruloplasmin and copper levels: Below normal Urinary copper: Raised Kidney biopsy: lupus nephritis Anti‐DNA and anti‐PCNA: Positive	Steroids, cyclophosphamide, hydroxychloroquine, mycophenolate mofetil along with copper‐chelating therapy	Symptoms initially improved, but other neurological symptoms emerged after 2 years

Abbreviations: ANA, antinuclear antibody; anti‐dsDNA, anti‐double stranded DNA; anti‐PCNA, anti‐proliferating cell nuclear antigen; IV, intravenous; KF rings, Kayser‐Fleischer rings; LFT, liver function test; SLE, systemic lupus erythematosus; US, Ultrasound.

## 2 Case History and Examination

A 17‐year‐old girl with no known comorbid condition came to the Civil Hospital Karachi with complaints of fever on and off for 4 months, yellow discoloration of skin and sclera for 20 days, and odd behavior and irrelevant talk for the past week. The patient was alright 4 months back when she started to develop a fever, intermittent and undocumented, relieved by taking antipyretics; there is no specific time for fever; it was associated with rigor, chills, body ache, and joint pain but not accompanied by sore throat, burning micturition, cough, and loose stools. For the last 20 days, the patient has been complaining of yellow discoloration of the sclera that progressed to the whole body associated with dark‐colored urine, accompanied by a purplish rash all over the body not related to pale stool, itching, abdominal pain, melena, or hematemesis. A few days later, she developed odd behavior with irrelevant talk but remained conscious without any neurological symptoms. Past medical history was insignificant. She has taken multiple antipyretics prescribed by her local GP—no significant transfusion history. Personal history is positive for disturbed sleep and decreased appetite.

## 3 Investigation, Diagnosis, and Treatment

**FIGURE 1:**
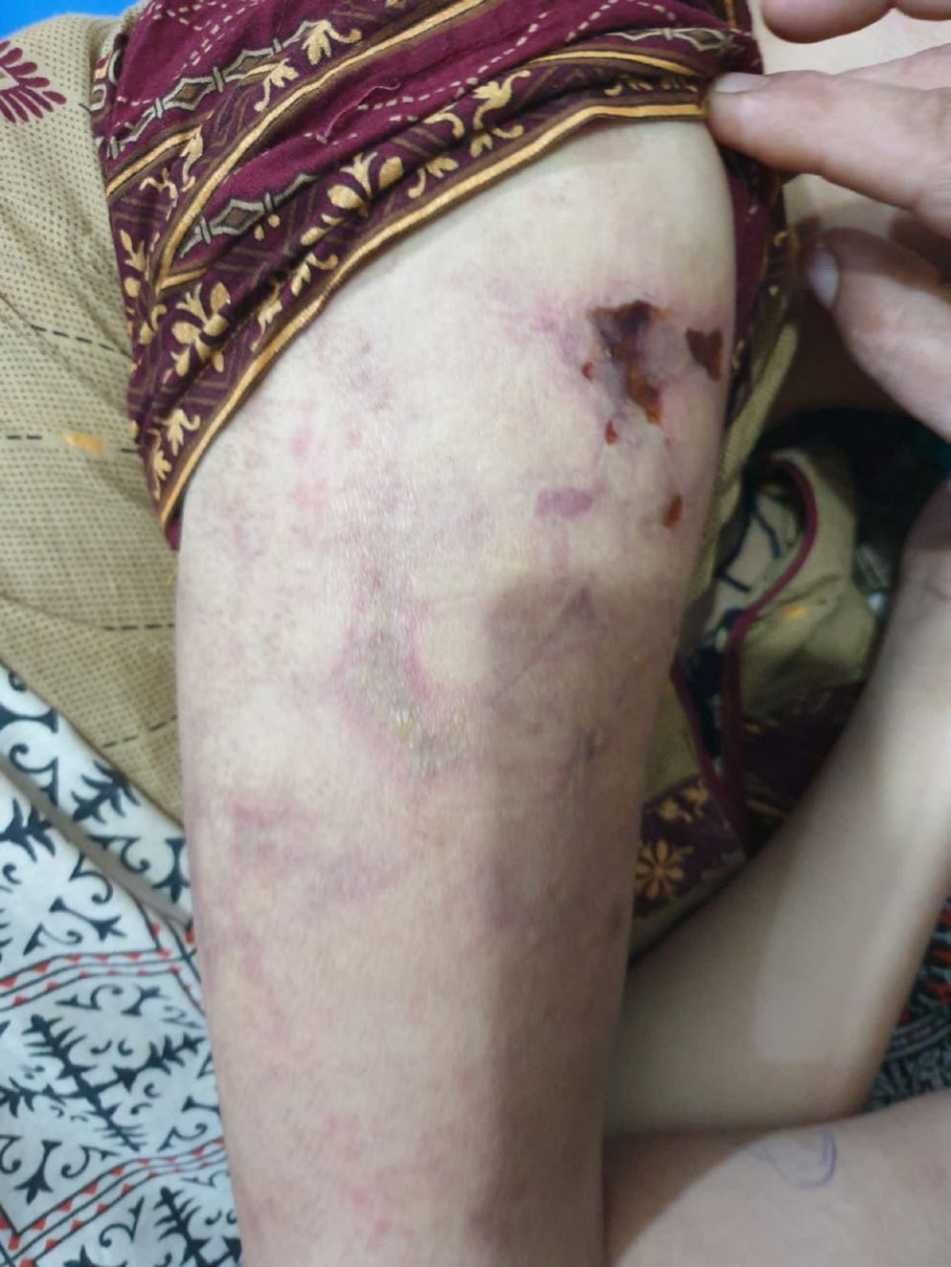
Purplish Rash And Reticular Pattern With Necrotic Plaques.

**FIGURE 2:**
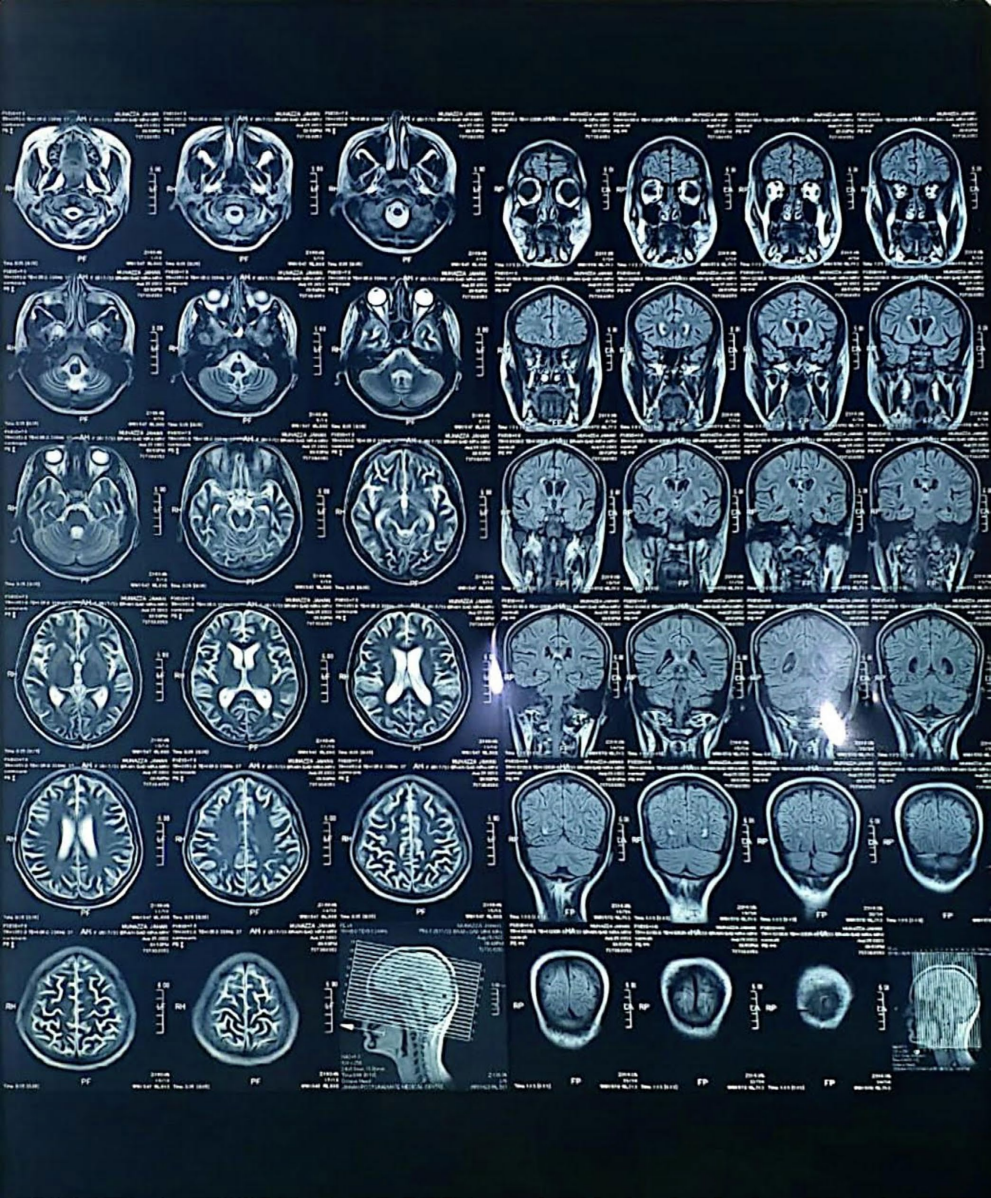
MRI Of the Brain Revealing A Dilated Ventricular System With Minimal Subependymal Seepage.

On initial examination, she looked sick and jaundiced, a young female average‐built conscious but disoriented with time, place, and person. She had a blood pressure of 100/60 mm of hg, pulse rate of 113 bpm, regular with a normal volume respiratory rate of 20 bpm. She had severe anemia, deep jaundice, mild dehydration, leukonychia, a puffy face with bilateral non‐tender parotid enlargement, oral ulcers, and angular stomatitis. Furthermore, on skin examination, the purplish rash looks like a reticular pattern with few necrotic plaques present on multiple sites (Figure 1). Except for the irrelevant talk, other neurological examinations were normal, and abdominal examination showed mild hepatosplenomegaly. The rest of the examinations were unremarkable. She is the only child of her consanguineous parents. The father was on antipsychotic medication for 20 years. Following admission, routine tests showed anemia (Hb = 6.6 mg/dL; normal = 12–16 mg/dL), leukopenia (TLC = 2; normal = 4–11 × 10^9^/L), thrombocytopenia (platelets = 40; normal = 150–450/mcL), elevated LDH (1027; normal = 140–280 U/L), decreased albumin (2.2; normal = 3.4–5.5 g/dL), raised globulin (4.4; normal = 2–3.5 g/dL), elevated total bilirubin (11.5; normal = 0.2–1.2 mg/dL), and SGOT (1292; normal = 8–45 units/L). Urine DR showed hematuria (8 − 10/hpf) and trace protein. Other lab findings, including thyroid profile, iron profile, HbA1C, and uric acid, were within normal ranges. Virologic tests were normal or negative; ANA titers were > 7.5 U/mL (normal = < 1.2 U/mL), anti‐dsDNA titers were 95.4 (normal = < 20 IU/L), RA factor > 20 IU/mL, and direct Coombs polyspecific 2+. C3 and C4 levels were low, and anti‐Ro/SSA antibodies were positive. Her serum ceruloplasmin was 0.2 (normal = 0.2–0.3 g/L), and 24‐h urine copper was 204 (normal < 60 micrograms/day). Ultrasound and Fibroscan were performed, revealing a normal ultrasound and a Fibroscan result of 3 kPa, with no indication of liver cirrhosis at diagnosis. Brain MRI showed a dilated ventricular system with minimal subependymal seepage (Figure 2), while MRA and MRV were unremarkable. EEG showed diffused 7–8 Hz alpha waves. Additional liver parameters, kidney function, and lab findings are detailed in Table 2.

**TABLE 2:** Laboratory investigations.

Hb	6.6 mg/dL
TLC	2 × 10^9^/L
Plt	40/mcL
Serum Albumin	2.2 g/dL
Globulin	4.4 g/dL
LDH	1027 U/L
Ferritin	452 μg/L
Serum ceruloplasmin	0.2 g/L
24 h urinary copper	204 μg/day
PT	10.5
INR	1.18
Total bilirubin	11.5 mg/dL
SGOT	1292 U/L
SGPT	161 U/L
ALP	261 U/L
GGT	346 U/L
A/G ratio	0.39
BUN	9 mg/dL
Cr	0.7 mg/dL
Na	136 mEq/L
K	3.7 mEq/L
Cl	107 mEq/L
Ca	7.2 mEq/L

Based on the laboratory findings of hemolytic anemia, lymphopenia, leukopenia, serositis, positive ANA, and anti‐dsDNA antibodies, the diagnosis of SLE was established according to the American College of Rheumatology revised criteria for the classification of SLE (Table 3). According to Leipzig criteria (Table 4), which include neurological symptoms, low serum ceruloplasmin, and increased 24‐h urinary copper, A score of 4 establishes a diagnosis of WD. Further findings suggested that the final diagnosis for this patient was acute hepatitis due to WD with secondary SLE. We initiated treatment with Slumedrol at a dose of 1 g for 5 days, alongside Rapricort at 1 mg/kg with a tapering schedule over 5 days. Additionally, Cap trientine 300 mg and zinc acetate 50 mg were administered twice daily. On day two, the patient developed seizures with a GCS drop to 11/15, leading to the initiation of Levetiracetam 500 mg twice daily for seizure control. Despite these efforts, the patient, unfortunately, did not survive, due to severe infection caused due to immunosuppressed state secondary to her autoimmune conditions, with trientine and zinc acetate continued until her passing.

**TABLE 3:** New ACR and EULAR criteria for the diagnosis of systemic lupus erythematosus (SLE).

Clinical domains points	Clinical domains points
**Constitutional domain**	13 Leukopenia
	3 points
Fever	14 Thrombocytopenia
2 points	4 points
**Cutaneous domain**	15 Autoimmune hemolysis
2 Nonscarring alopecia	4 points
2 point	**Renal domain**
3 Oral ulcers	16 Proteinuria > 0.5 g/24 h
2 points	4 points
4 Oral ulcers	17 Class 2 or 5 lupus nephritis
2 points	8 points
5 Subacute cutaneous or discoid lupus	18 Class 3 or 4 lupus nephritis
2 points	10 points
6 Acute cutaneous lupus	**Immunologic domain**
6 points	19 Anticardiolipin IgG > 40 GPL or Lupus anticoagulant
**Arthritis domain**	2 points
7 Synovitis in at least 2 joints or tenderness in At least 2 joints and at least 30 min of Morning stiffness	
6 points	20 Low C3 or low C4
**Neurologic domain**	3 points
8 Delirium	21 Low C3 and low C4
2 points	4 points
9 Psychosis	**Highly specific antibodies domain**
3 points	22 Anti‐dsDNA antibodies
10 Seizures	6 points
5 points	23 Anti‐smith antibodies
**Serositis domain**	6 points
11 Pleural or pericardial effusion	
5 points	
**Hematologic domain**	
12 Acute pericarditis	
6 points	

*Note:* All patients classified as SLE must have a serum ANA titer of at least 1:80 on human epithelial 2 positive cells or an equivalent positive cell. In addition, a patient must have at least 10 points from these criteria.

**TABLE 4:** Leipzig criteria for the diagnosis of Wilson's disease.

Clinical or laboratory findings		Points
Kaiser fleischer rings	Present	2
	Absent	0
Neurological symptoms or MRI findings	Severe	2
	Mild	1
	Absent	0
Serum ceruloplasmin levels (g/L)	< 0.1	2
	0.1–0.2	1
	> 0.2	0
24‐h urinary copper	> 2× upper limit of normal	2
	1–2× upper limit of normal	1
	Normal	0
	Normal, but > 5× upper limit of normal after D‐penicillamine	2
Coombs‐negative hemolytic anemia	Present	1
	Absent	0
Total liver copper level (micromole/g)	> 5× upper limit of normal (> 4)	2
	Increased (0.8–4)	1
	Normal (< 0.8)	−1
	Rhodamine‐positive granules present	1
Genetic mutation	Present on both chromosomes	4
	Present on one chromosome	1
	Absent	0
Total score	Diagnosis established	4
	Diagnosis likely	3
	Diagnosis unlikely	2 or less

## 4 Conclusions and Results

In conclusion, the co‐occurrence of WD and SLE presents a rare and complex diagnostic challenge due to overlapping symptoms, particularly neuropsychiatric and renal manifestations. This case highlights the importance of careful differential diagnosis and individualized treatment strategies when managing patients with multiple systemic conditions. Despite initial therapeutic improvement, the unfortunate outcome due to a blood transfusion reaction underscores the need for close monitoring of potential complications in such complex cases. Clinicians should maintain a high index of suspicion for autoimmune diseases in patients with WD, especially when they present with atypical symptoms.

## 5 Discussion

Mucormycosis is an aggressive fungal infection, often associated with significant morbidity and mortality. This case is unusual as the patient had no predisposing factors like diabetes or immunosuppression. Trauma is an infrequent trigger for mucormycosis, but it likely provided an entry point for the Rhizopus species in this case. This case demonstrates that early surgical intervention and antifungal therapy are crucial for favorable outcomes. This report reinforces the need for clinicians to consider mucormycosis even in immunocompetent pediatric patients presenting with unexplained ocular symptoms post‐trauma.

This case is exceptional in that there are no predisposing factors, highlighting the fact that even immunocompetent people can get this potentially fatal infection after a minor injury.      A similar case was reported in another study in which a 31–year–old male underwent dental surgery, developed necrotizing lesions, and was later on diagnosed with mucormycosis [3]. The dental profession is responsible for being aware of the possibility of potentially severe and potentially fatal complications in healthy individuals [4]. The use of corticosteroids is also a risk factor for mucormycosis. The high death rate, even with improvements in diagnoses, emphasizes the necessity of greater awareness and prompt action in at‐risk groups [7]. In healthy hosts, neutrophils that destroy hyphal components by oxidative burst and macrophages that prevent spore germination are the defensive mechanisms. Infections in diabetics are caused by macrophage dysfunction [8]. Another study shows that GI mucormycosis can occur in malnourished and immunocompetent patients, so if malnourished patients exhibit abdominal symptoms and risk factors, clinicians should consider GI mucormycosis [9]. In this case, the symptoms of the patient were suggestive of rhabdomyosarcoma. The diagnosis was confirmed on histopathology. a study says that in immunocompetent patients' Nonspecific symptoms make diagnosis difficult; biopsy and culture are required [1]. In immunocompromised patients with liver cirrhosis, posaconazole can be used safely and effectively to treat mucormycosis [10].

Though rare, an association between WD and SLE has been reported, with the underlying mechanisms still not fully understood [10]. Antos et al. [11] note that SLE induced by D‐penicillamine can also manifest in patients undergoing treatment for WD. This case underscores the diagnostic complexity of WD, particularly when overlapping autoimmune conditions are present [5]. The patient's initial presentation with jaundice and anemia, followed by neurological symptoms, underscores the importance of considering WD in the differential diagnosis, particularly in the presence of liver dysfunction and autoimmune markers. Understanding the rare association between WD and SLE could provide insights into the pathophysiology of these conditions and improve management strategies [3].

## Author Contributions

**Suresh Bhagoowani:** supervision, writing – original draft, writing – review and editing. **Uooja Devi:** writing – original draft, writing – review and editing. **Aqsa Munir:** writing – original draft, writing – review and editing. **Ummulkiram Hasnain:** writing – original draft, writing – review and editing. **Saad Khan:** writing – original draft, writing – review and editing. **Javed Iqbal:** writing – original draft, writing – review and editing. **Tehseen Akhtar:** supervision, writing – review and editing.

## Ethics Statement

Ethical approval does not imply to case reports from institutional IRB.

## Consent

Written informed consent was obtained from the patient to publish this report in accordance with the journal's patient consent policy.

## Conflicts of Interest

The authors declare no conflicts of interest.

## Data Availability

The authors have nothing to report.
